# Indents

**Published:** 1919-06

**Authors:** 


### INDENTS

☞**Brief Articles of Technical or Scientific Value will receive notice and comment. Contributions invited.**

---

An Iodine Lotion. To eighty parts of Benzol add ten parts of iodine crystals and ten parts of menthol crystals. Keep well corked as it is a volatile preparation.—N. Rudin, in *Dental Digest.*

---

Anesthetizing and Sterilizing Gum Locally. To nine parts of tincture of iodine add one part of benzol. Use as a local application to sterilize and obtund the gum when injecting for local operations.—F. J. Crymes, in *Dental Digest.*

---

To Duplicate Plaster Casts. Soak about 150 leaves of common gelatin in cold water for one or two hours, gradually adding four or five ounces of oil, constantly stirring. Place the cast in an enameled vessel and pour the above mixture over it. After about two hours the mixture will have hardened, when the cast may be poured.—F. A. B., *Dental Office Laboratory.*

Conforming the Wax for Denture Casting. After oiling the master cast—which should be made from Weinstein's stone or Spence plaster—with a light oil such as alboline, the thin wax is carefully adapted to the cast. Care must be used to prevent the wax from breaking or stretching. By using a warm, wet napkin or cheese cloth for the conforming of the wax, there will be no breaking or lack of adaptation of the wax, and the work can be done in one-half of the usual time.—F. W. Frahm, in *Pacific Gazette*.

---

To Pass Strip Through a Tight Contact. When it is difficult to pass a finishing strip into the inter-proximal space on account of a tight contact point, sometimes it can be done by first passing a ligature to the tightest point. Then pass the strip down against it and carry both ligature and strip through.—J. F. Nelson, *Oral Health.*

---

Perry Universal Separator. As for separators, medium separation is all that is necessary, and there is only one separator, and you haven't got it—the Perry Universal Separator. The Perry Universal Separator takes hold of the teeth and pulls them apart; it does not wedge them apart and wedge the gum at the same time, as the horse killers do.—A. E. Webster, *Dominion Dental Journal.*

---

Pepsodent. The keynote of the "Pepsodent" advertising is the claim that, used as a dentifrice, it will digest or dissolve the dental mucin plaques through the action of pepsin which has been incorporated in the paste. The acid medium necessary for the activation of the pepsin is claimed to be acid calcium phosphate; the abrasive substance said to be used is tricalcium phosphate. Pepsodent has been exhaustively studied by Dr. William J. Gies and his collaborators in the Biochemical Laboratory of the Schools of Medicine and Dentistry of Columbia University. Dr. Gies in a letter published in *The Journal,* April 28, 1917, briefly summarizes the findings of this investigation. Regarding the alleged digestive powers of Pepsodent, Dr. Gies wrote:

"We have found that none of these digestive claims is warranted in any degree. 'Pepsodent' is devoid of the digestive power on dental mucin plaques that is commercially ascribed to it. Mucin plaques can not be digested from teeth by any advertised use of 'Pepsodent.' There is about as much common sense in the proposed application of 'Pepsodent' for this purpose as there is in the oral administration of a few grains of lactopeptin to improve impaired tryptic digestion in the intestine."

A complete report of the investigation of Dr. Gies and his collaborators appeared in *The Journal of the Allied Dental Societies,* September, 1917. The investigation showed that the acidity of Pepsodent is due largely, if not entirely, to acid calcium phosphate. The claim by the Pepsodent concern that the use of this preparation stimulates the production of saliva is true. It does stimulate the flow of saliva to an extent that speedily neutralizes the very feeble acid present in Pepsodent. Thus, any possible influence that this "acid" might otherwise exert is, as Dr. Gies says, "promptly and completely nullified." As to whether the abrasive used in Pepsodent is injurious to the teeth, Dr. Gies reported that it appeared "to be devoid of harmful mechanical influence on dental enamel" in the ordinary use of the preparation as a dentifrice. He added: "We are not positive on this point, however."—*Journal A. M. A.,* May 10, 1919.

---

Root-Resection. Root-resection should not be attempted if the bone is absorbed over more than the apical third of the root, but such a tooth should be extracted and the socket curetted.—Earle H. Thomas, *Dental Review.*

Method for Retaining Dentures in Flat Hard Surfaces. For lower dentures make a groove about the thickness of an ordinary pin in depth and width around the model before packing about a millimetre from the edge of plate when finished, then after the flask is packed cut a strip of unvulcanized velum rubber about a millimetre wide and carefully cover the groove—by using a little chloroform on the rubber it may be made to stick to plaster model; when properly completed a narrow ridge of soft rubber will come in contact with patient's gum tissue and form a cushion and suction. A similar proceeding may be used on upper case, but I have found a groove around the air chamber treated as above quite satisfactory. Another method is to pack a space two or three millimetres wide all around the ordinary air chamber. A lip can be made on this soft rubber around the air chamber by bevelling the edge of the air chamber metal and fastening on mold over a sheet of soft rubber cut quarter inch larger than metal and a hole cut slightly smaller than metal. Using above methods there is no need of failure in retaining dentures.—Captain Teetzel, in *Oral Health.*

---

Poor Impressions. I have for many years assisted prominent dentists in the construction of difficult denture cases and, until recent laws prohibited, always secured my own impresions, and the result of my efforts gave satisfaction to the dentist as well as the patient. Now, however, I am compelled to use the impressions and casts of dentists who in many cases have not even learned the rudiments of impression taking, with the result that ofttime my laboratory is blamed for failure when the real cause is lack of knowledge on the part of the dentists themselves. Ask any dental laboratory if this is not so. —A New Jersey Laboratory Operator.

